# Diverting Ileostomy Duration Is the Main Determinant of Ileostomy-Related Complications after Surgical Treatment of Rectum Cancer

**DOI:** 10.1155/2020/4186857

**Published:** 2020-04-07

**Authors:** Nadir Adnan Hacim, Ahmet Akbas, Serhat Meric, Yüksel Altinel, Onder Karabay, Erkan Yavuz

**Affiliations:** ^1^Department of General Surgery, Bagcilar Training and Research Hospital, Istanbul, Turkey; ^2^Surp Pırgiç Armenian Hospital, Istanbul, Turkey

## Abstract

**Background:**

This study aimed to investigate factors associated with the development of ileostomy complications in rectal cancer patients, including those who received neoadjuvant treatment.

**Methods:**

This retrospective trial included 133 consecutive patients who underwent surgery for rectal cancer with temporary diverting ileostomy. Patients' demographic characteristics as well as the pre- and postclosure outcomes and complications were analyzed.

**Results:**

In logistic regression analysis, longer duration of ileostomy emerged as a significant independent predictor of any complication during ileostomy. The respective odds ratios for 3–6 months and >6 months vs. <3 months of ileostomy duration were as follows: OR, 4.5 (95% CI, 1.2–16.7), *p*=0.023; and OR, 15.2 (95% CI, 3.1–75.2), *p*=0.001. An additional stepwise model also identified hypertension as a significant predictor. In stepwise logistic regression model, adjuvant chemoradiotherapy emerged as significant independent predictor of “any ileostomy-related complication after ileostomy closure”: OR, 4.5 (2.0–10.2), *p* < 0.001.

**Conclusion:**

Duration of ileostomy appears to be the main determinant of ileostomy-related complications. Patients who had received neoadjuvant or adjuvant therapy had longer ileostomy duration, which may be attributed to the concerns of the surgeon or to the complications themselves.

## 1. Introduction

Globally, colorectal cancer is the third common cancer in women and second in men, with an increasing incidence [1]. Accordingly, more than 1.8 million new cases of colorectal cancer have been diagnosed in the year 2018 worldwide, and 881,000 deaths have been recorded in the same year [1]. Colorectal cancer originates from the rectum in nearly 30% of the patients. Current strategies based on multidisciplinary management with definitive preoperative tumor staging, improved surgical techniques, neoadjuvant chemoradiation therapy, and adjuvant therapy have resulted in improved disease-free survival and overall survival rates in patients with rectal tumors [2]. In surgical practice, total mesorectal excision (TME) is the standard surgical procedure for patients with advanced rectal tumors [2]. The most significant complication of TME is the leakage of colorectal anastomosis, which has been reported to occur in 3% to 19% of the cases [3].

Although diverting ileostomy can mitigate the severity of the consequences of an anastomotic leak, various morbidities related to stoma can occur [3, 4]. The reported incidence of stoma-associated morbidities varies between 17% and 45% [3–5]. In addition to perioperative complications (obstruction, stoma site infection, parastomal hernia, electrolytic imbalance, etc.), it can also lead to psychological distress through distorted self-image. Furthermore, these patients have been reported to have an impaired quality of life [4]. Thus, although the closure of ileostomy as early as possible after surgery is a priority for both the surgeons and patients alike [4, 5], a delay is not uncommon, mainly due to risk factors such as complications at the site of anastomosis or adjuvant treatment of cancer [6]. Additionally, pelvic irradiation (with neoadjuvant radiotherapy) imposes further risks and the terminal ileum may be exposed to irradiation; thus, neoadjuvant treatment may increase the risk of complications at the site of ileostomy [6, 7].

Currently, no consensus exists regarding optimal timing for the closure of ileostomy in patients who underwent surgery for rectal cancer, particularly for those who received neoadjuvant chemoradiotherapy. Surgeons display significant variability with regard to the practice of stoma reversal surgery [3, 8]. In this study, we aimed to assess the factors that have an impact on ileostomy-related complications in rectal cancer patients, including those who previously received neoadjuvant treatment.

## 2. Methods

### 2.1. Patients

Patients that underwent surgical resection (with or without neoadjuvant treatment) with temporary ileostomy for rectum cancer between February 2013 and October 2018 were included in this retrospective study. We excluded patients who had coexisting inflammatory bowel diseases (ulcerative colitis and Crohn's disease). Demographical and clinical parameters were extracted from patient files. Factors affecting ileostomy-related and colonic anastomosis-related complications as well as overall survival were examined.

### 2.2. Outcome Measures

Outcome measures were complications potentially related with ileostomy, either during its presence or after its closure. Development of following ileostomy site-related complications, metabolic complications, and distal anastomosis-related complications when the ileostomy was in place were examined: stomal stenosis/obstruction, retraction (displacement below 1 cm skin level), skin irritation, ischemia, parastomal infection (observed erythema, swelling, pus discharge), parastomal hernia/prolapses, acute renal failure, electrolyte imbalance (hyponatremia or hypokalemia), stenosis of colorectal anastomosis, leakage of colorectal anastomosis, and intraabdominal abscess related to colorectal anastomosis (pelvic abscess). In addition, four combined outcome variables were examined: “any ileostomy site-related complication,” “any distal anastomosis-related complication,” “any metabolic complication,” and “any complication during ileostomy”. The examined postileostomy closure complications were as follows: postclosure obstruction of ileum, leakage, stoma site infection, reoperation, intraabdominal abscess (ileostomy related), diarrhea, and a combined complication variable “any complication after ileostomy closure.” Survival data were extracted from patient files or obtained by phone contact. All complications were also classified using Clavien–Dindo grading system from grade I to grade V [9].

### 2.3. Statistical Analysis

Data were analyzed using IBM SPSS Statistics version 21.0 software (SPSS Inc., Chicago, IL). Descriptive data are presented in number (percentage), mean ± standard deviation, and median (range), where appropriate. Depending on normality of data and number of groups, continuous variables were compared using Student's *t*-test, one-way ANOVA, Mann–Whitney *U*, or Kruskal–Wallis test. Categorical variables were compared using Pearson's chi-squared test or Fisher's exact test. A logistic regression model was used to identify significant independent predictors of complication outcomes. Overall survival was defined as the time elapsed between surgical treatment and death. Survival was estimated using Kaplan–Meier analysis and intergroup comparisons were performed using log-rank test. A *p* value < 0.05 was considered the indication for statistical significance.

## 3. Results

### 3.1. Patient Characteristics

Table 1 shows demographic and clinical characteristics of all patients. The great majority of the patients had adenocarcinoma, and almost half were at stage I. More than half received neoadjuvant therapy (52.6%), and almost half received adjuvant therapy (46.6%). Mean duration of ileostomy was 6.4 months; and 41 (30.8%), 20 (15.0%), and 72 (54.2%) patients had ileostomy for <3 months, 6–9 months, and >6 months, respectively.

### 3.2. Predictors of Complications during Ileostomy

In univariate analysis of potential factors, presence of hypertension, stages II-III disease, neoadjuvant treatment (either chemotherapy or radiochemotherapy), adjuvant treatment (either chemotherapy or radiochemotherapy), and longer duration of ileostomy (>3 months) were associated with more frequent combined outcome of “any ileostomy-related complication during ileostomy.” Multivariate analysis with routine logistic regression identified only longer durations of ileostomy as a significant independent predictor of any complication during ileostomy. When compared to <3 months duration, 3–6 months and >6 months ileostomy durations had the following odds ratios: OR, 4.5 (95% CI, 1.2–16.7), *p*=0.023; and OR, 15.2 (95% CI, 3.1–75.2), *p*=0.001. However, when stepwise logistic regression model was applied, hypertension also emerged as significant independent predictor of “any complication during ileostomy”: OR, 2.6 (1.2–6.0), *p* < 0.021.

### 3.3. Predictors of Complications after Ileostomy Closure

In univariate analysis of potential factors, stages II-IV disease, neoadjuvant treatment, adjuvant radiochemotherapy, and longer duration of ileostomy (>6 months) were associated with more frequent combined outcome of “any ileostomy-related complication after ileostomy closure.” None of the factors emerged as significant independent predictor of “any ileostomy-related complication after ileostomy closure” on routine logistic regression. However, when stepwise logistic regression model was applied, adjuvant chemoradiotherapy emerged as significant independent predictor of “any ileostomy-related complication after ileostomy closure”: OR, 4.5 (2.0–10.2), *p* < 0.001.

### 3.4. Ileostomy Duration and Complications

Table 2 shows the comparison of each individual complication or combined complication variables by the duration of ileostomy. Frequencies of a number of complication variables are higher among patients who had ileostomy longer than 3 months and ileostomy duration more than 6 months seem to pose even high risk for several of these variables. In addition, duration of hospitalization at the time of ileostomy closure was significantly longer in patients that had >6 months of ileostomy when compared to patients that had <3 months of ileostomy (8.1 vs. 5.1 days, *p* < 0.001).  Table 3 shows the grading of the complications based on Clavien–Dindo grading system. The number of complications in each grade was significantly higher as time interval before and after the ileostomy closure increased (*p* < 0.05 for all) (Table 3).

Despite the relatively low frequencies, multivariate analysis for individual complications was done. Other than significant associations with ileostomy duration, only presence of hypertension emerged as a significant predictor of several outcomes: any metabolic complication (OR, 9.3 (1.2–7.8), *p*=0.032) and obstruction after ileostomy closure (OR, 6.4 (1.6–26.3), *p*=0.010).

### 3.5. Associations with Neoadjuvant and Adjuvant Treatments

Although univariate analyses identified associations with these factors and increased risk for certain complication outcomes, such associations did not persist on multivariate analyses. On the other hand, ileostomy durations were longer for patients who received neoadjuvant and adjuvant treatments. For neoadjuvant treatment, it was 270.5 versus 110.7 days (*p* < 0.001), when compared to patients that did not receive neoadjuvant. For adjuvant treatment, it was 267.8 versus 111.2 days (*p* < 0.001), when compared to patients that did not receive adjuvant treatment.

### 3.6. Associations between Ileostomy Duration and Survival

Survival data were available for 125 patients with a mean follow-up duration of 25.3 ± 12.4 months. Mean overall survival was similar across three different groups of ileostomy duration: 52.7 ± 4.3, 51.0 ± 4.9, and 45.1 ± 2.9 months for <3 months, 3 to 6 months, and >6 months ileostomy duration, respectively.

## 4. Discussion

In patients undergoing surgery for low-lying rectal cancer, diverting ileostomy reduces the frequency and severity of colorectal anastomotic complications, although it may itself lead to serious morbidities. Concurrent chemoradiotherapy before or after rectal surgery may also elevate the risk of ileostomy-related complications. In this study, we observed a significant impact of delayed ileostomy reversal (more than 6 months in particular) on ileostomy-related morbidity. To our knowledge, this study represents one of the few published studies that also examined the effects of both neoadjuvant and adjuvant treatments on complications after ileostomy closure in rectal cancer patients.

Not only adjuvant chemoradiotherapy but also neoadjuvant treatments appear to have adverse effects on both colonic and small bowel anastomotic integrity, and both of these therapeutic approaches may delay the ileostomy closure through an increased rate of colorectal and ileostomy complications [2, 5, 8, 10]. However, factors such as the use of different radiotherapy regimens in different centers have generally precluded a clear-cut consensus regarding the extent of such adverse effects. In our study, a radiation dose of 40 to 50 Gy was administered to patients, and ileostomy duration was longer for patients who received neoadjuvant and adjuvant treatments. These observations are consistent with the results of some previous pilot studies [2, 10–13] and might reflect the reluctance of surgeons for taking the risks associated with anastomotic leaks [14]. Also, one other potential explanation might be the high incidence of ileostomy-related complications in patients who also receive irradiation and chemotherapy. Such complications may prevent a timely closure in certain patients [15]. Leakage of the colonic anastomosis represents the most significant complication among others, which was reported in 13.9% of the cases in a study by Phatak et al. [14]. Several factors other than chemoradiotherapy may affect the leakage risk in colonic anastomoses including the method of anastomosis, skill and expertise of the surgeon, and the level of anastomosis. In the current study, rectal surgery was performed by the same surgical team using the same procedures. In our series, early and delayed reversal groups exhibited some differences in terms of the rate of anastomotic leakage (2.4% for < 3 months, 10% for closure between 3 and 6 months, and 12.5% for > 6 months), although the observed differences were insignificant. It appears that the leakage of the colonic anastomosis may be the single most important reason for surgeons to avoid early closure of a stoma.

Another point to consider in these patients is the duration of ileostomy and its effects on ileostomy-related complications. In this regard, several studies focused on early and delayed ileostomy reversal time and investigated the impact of the timing of closure on morbidity and mortality rates [1, 4, 10, 11, 15–17]. Rubio-Perez et al. [4] and Danielsen et al. [16] reported that delayed closure contributed to high rates of ileostomy site-related complications. Similarly, a randomized clinical study [15] and a large comprehensive meta-analysis [11] showed lower rates of medical and surgical complications (especially small bowel obstruction) in the early closure group. In our findings, frequencies of a number of complication variables are higher among patients who had ileostomy longer than 3 months and ileostomy duration more than 6 months. Ileostomy-site complications were seen in 61.1% of the patients who had delayed closure as compared to 17.1% and 35% in early (<3 months) and in intermediate closure (between 3 and 6 months) groups, respectively. These observations are in line with some previous studies [5, 10, 15–17] all showing higher rates of complications with increasing stoma duration and all advocating early stoma closure for the prevention of complications. This is particularly relevant, since complications such as peristomal dermatitis, electrolyte abnormalities, bowel obstruction, acute renal failure, dehydration, and parastomal hernia may delay the initiation of adjuvant chemoradiotherapy. On the other hand, delayed stoma closure itself may be associated with significant negative impact on quality of life, through its effects on sexuality, body image, stress levels, and the presence of a stoma bag [17, 18].

Certainly, the timing of ileostomy closure should be determined according to each patient's clinical status. For instance, a good metabolic status and an uneventful postsurgical period represent important indications for ileostomy closure. An early closure of stoma may be a viable option for patients who have no clinical or radiological sign of anastomotic leakage or stiffness after a careful assessment, and it will help alleviate the social, physical, and psychological burden of stoma [5, 18]. However, it should also be noted that some authors found no effect of early closure of temporary ileostomy on health-related quality of life indices [19].

As it was confirmed by a previous study [8], both preoperative and postoperative radiotherapy may be risky for stoma closure. In our opinion, it will be safe to keep the stoma for a while longer in such patients after taking the essential measures.

Certain limitations of our study should be mentioned. First, this was a retrospective review with relatively small number of cases in a single center. Due to retrospective design of the study, we could not analyze smoking and alcohol usage because of incomplete data. In addition, impact of the overall health status of the patients on decision about the timing of ileostomy closure was not analyzed. Desire of the patients for early closure of ileostomy, necessity of adjuvant treatment causing a delay for closure, and different approaches of the surgeons for closure of ileostomy might be the other variables on the timing of the closure. Therefore, both the patient- and surgeon-related factors might cause selection bias in the study. Some analyses may not have had adequate statistical power to show statistical significance in comparisons. Also, since some of our cases were foreigners (mostly from Syria) or resided in distant locations in Turkey, ileostomy-related complications could not be followed up in these subjects.

In conclusion, duration of ileostomy seems to be the main determinant of ileostomy-related complications. Of particular note was the longer duration of ileostomy in patients who received neoadjuvant or adjuvant therapy, which may be attributed to the concerns of the surgeon regarding the higher likelihood of complications among these patients or to the complications themselves. Therefore, it seems plausible to close the ileostomy as early as possible if the condition of the patient allows. Further prospective studies with larger and more homogenous patient samples will shed more light on this issue, particularly with respect to the identification of differences among subgroups in terms of complications related to ileostomy or distal anastomosis.

## Figures and Tables

**Table 1:** Patient characteristics.

Characteristic	*n* = 133
Age, y, mean ± SD (median, range)	61.3 ± 13.5 (63, 32–87)

Male gender	82 (61.7%)

BMI (kg/m^2^)	27.87 ± 5.70

Preoperative condition	
ASA score	
1	69 (51.9%)
2	41 (30.8%)
3	19 (14.3%)
4	4 (3%)

Hemoglobin, g/dL, mean ± SD	12.6 ± 4.1

Albumin, g/dL, mean ± SD	4.1 ± 0.9

Diabetes	37 (27.8%)

Hypertension	67 (50.4%)

Tumor characteristics	

Histological type	

Adenocarcinoma	121 (91.0%)

Mucinous adenocarcinoma	10 (7.5%)

Signet ring cell carcinoma	1 (0.8%)

Neuroendocrine tumor	1 (0.8%)

Tumor diameter, cm (mean ± SD)	4.5 ± 1.5

Stage	
I	63 (47.4%)
II	19 (14.3%)
III	44 (33.1%)
IV	7 (5.3%)

Grade	
I	73 (54.9%)
II	44 (33.1%)
III	15 (12.0%)

Distance from anal verge, cm, mean ± SD	8.0 ± 2.7

Neoadjuvant treatment	
No neoadjuvant treatment	63 (47.4%)
Neoadjuvant radiotherapy	26 (19.5%)
Neoadjuvant radiochemotherapy	44 (33.1%)
Neoadjuvant-surgery duration,^*∗*^ days, mean ± SD, (median, range)	79.1 ± 42.0 (75.5, 9–201)

Adjuvant treatment	
No adjuvant treatment	62 (46.6%)
Adjuvant chemotherapy	22 (16.5%)
Adjuvant radiochemotherapy	49 (36.8%)

Duration of ileostomy, days, mean ± SD, (median, range)	194.8 ± 120.0 (195, 16–670)

Unless otherwise stated, data are presented in frequency (percent). ^*∗*^Duration between the end of neoadjuvant treatment and surgery in patients that received neoadjuvant treatment.

**Table 2:** Comparison of complication frequencies by the duration of ileostomy.

Complication	<3 months *n* = 41	3–6 months *n* = 20	>6 months *n* = 72	*p*
Complications during ileostomy				
Ileostomy site related				
Stomal stenosis/obstruction	0	0	11 (15.3%)	0.006
Retraction	1 (2.4%)	1 (5.0%)	9 (12.5%)	0.148
Skin irritation	5 (12.2%)	3 (15.0%)	19 (26.4%)	0.160
Ischemia	1 (2.4%)	0	18 (25.0%)	0.001
Parastomal infection	0	1 (5.0%)	20 (27.8%)	<0.001
Parastomal hernia/prolapsus	0	2 (10.0%)	5 (6.9%)	0.166
Any ileostomy site-related complication	7 (17.1%)	7 (35.0%)	44 (61.1%)	<0.001
Distant anastomosis related				
Leakage at colorectal anastomosis	1 (2.4%)	2 (10%)	9 (12.5%)	0.197
Stenosis of colorectal anastomosis	1 (2.4%)	1 (5.0%)	14 (19.4%)	0.016
Intra-abdominal abscess (pelvic)	1 (2.4%)	3 (15.0%)	14 (19.4%)	0.039
Any distal anastomosis-related complication	2 (4.9%)	4 (20.0%)	27 (37.5%)	<0.001
Metabolic				
Acute renal failure	1 (2.4%)	1 (5.0%)	7 (9.7%)	0.315
Hypokalemia/hyponatremia	0	3 (15.0%)	8 (11.1%)	0.059
Any metabolic complication	1 (2.4%)	4 (20.0%)	14 (19.4%)	0.033

*Any complication during ileostomy*	10 (24.4%)	11 (55.0%)	57 (79.2%)	<0.001

Complications after ileostomy closure				
Obstruction (ileostomy anastomosis)	5 (12.2%)	2 (10%)	16 (22.2%)	0.258
Leakage at ileostomy	0	1 (5.0%)	7 (9.7%)	0.110
Stoma site infection	1 (2.4%)	3 (15.0%)	20 (27.8%)	0.003
Reoperation	2 (4.9%)	1 (5.0%)	6 (8.3%)	0.737
Intra-abdominal abscess (ileostomy related)	0	1 (5.0%)	9 (12.5%)	0.048
Diarrhea	3 (7.3%)	5 (25.0%)	10 (13.9%)	0.165

*Any complication after ileostomy closure*	11 (26.8%)	10 (50.0%)	45 (62.5%)	0.001

**Table 3:** Comparison of complication frequencies according to the Clavien–Dindo classification.

Clavien–Dindo classification	Complications during ileostomy, *n* (%)				Complications after ileostomy closure, *n* (%)			
	<3 months	3–6 months	>6 months	*p*	<3 months	3–6 months	>6 months	*p*
Grade I	7 (8.9%)	10 (12.8%)	17 (21.8%)		9 (13.6%)	5 (7.6%)	30 (45.5)	
Grade II	0	0	0		0	0	0	
Grade IIIa	3 (3.8%)	5 (6.4%)	27 (34.6%)		0	4 (6.1%)	9 (13.6%)	
Grade IIIb	0	0	0		2 (3%)	1 (1.5%)	6 (9.1%)	
Grade IV	1 (1.3%)	1 (1.3%)	7 (8.9%)		0	0	0	
Grade V	0	0	0		0	0	0	
Total	**11 (14.1%)**	**16 (20.5%)**	**51 (65.4%)**	**<0.001**	**11 (16.7%)**	**10 (15.2%)**	**45 (68.1%)**	**<0.001**

## Data Availability

The retrospective data used to support the findings of this study are available from the corresponding author upon request.
